# The Dublin Medical Press vs. Dr. Flint’s Prize Essay

**Published:** 1854-02

**Authors:** 


### EDITORIAL DEPARTMENT.

*The Dublin Medical Press* vs. *Dr. Flint's Prize Essay.*—The Dublin Medical Press of November 6, 1853, has a review of the essay on "Variations of Pitch in Percussion and other Respiratory Sounds, and their application to Physical Diagnosis," to which the National Medical Association, two years since, awarded a prize.

This essay, which on this side of the Atlantic has been supposed, by competent judges, to be a real and valuable addition to medical science, is met upon the other side, with that "damning with faint praise," and assumption of superior and earlier knowledge, which invariably returns from John Bulldom to greet the ears of the American discoverer.

We propose, in the absence of our senior, to give to the assumptions of the Dublin Medical Press such examination, as the real importance of the subject merits; and that our readers may have a full appreciation of the criticism under discussion, we shall, in this notice, introduce it in installments as they require to be met, but giving it, though divided, without subtraction, or change in the order of its sentences. We quote:

"This essay, which, it appears, was awarded a prize, consists mainly of a few observations on what the writer terms the pitch, that is, as we understand him, the received degrees of intensity of the sounds heard in percussion and respiration. Whilst we think the essay a good one, we must observe that the author has been forestalled in, we believe, every observation he has made. The pitch of the various sounds of both percussion and auscultation has been long familiar to us on this side the Atlantic. To the work of Walshe alone we need refer in proof; and though our author quoted this work, we think parts of it must have escaped his observation."

We prefer to consider this extract separately. First, in regard to the number of these observations spoken of as "a few." They comprise in all forty-one cases, a number sufficiently large when we consider the care with which they were made, the fact that the reality of the sounds was verified by other auscultations, that they include pneumonitis, pleuritis, gangrene and pneumothorax, small tuberculous deposit, abundant tuberculous deposit, tubercle advanced to excavation, and tubercle arrested; and that, wherever opportunity was afforded, they were verified by autopsy. Aside from these pathological observations, explorations of the chests of twenty-seven healthy individuals were made, with the view of ascertaining the normal variations in pitch. The whole number is, then, sixty-eight, as many, we have no doubt, as were ever made by a single person, to elucidate a single symptom.

Secondly, *we* do *not* understand the pitch of a sound to have anything to do with its intensity. Intensity is a contingent, and not an intrinsic quality. A very feeble sound may have the same pitch as a very intense one—at least on this side the Atlantic. The assertion of the "Press" as to this point, implies either deficient knowledge of the subject, or great carelessness in ascertaining the opinions of the author whom he criticises. The real issue is involved in the question as to whether or no Dr. Flint has been preceded by Walshe or others in the observations of these sounds.

Upon the first page of the Prize Essay is a *resume* of the present state of knowledge, in which Dr. Walshe is mentioned as noticing "variations in pitch, and several important facts connected with them. But he apparently loses sight of their practical applications, making no reference to them in connection with the diagnosis of individual thoracic diseases."

Before examining Walshe on this subject, let us inquire, what is an observation? If it consists merely in the hearing of certain sounds, and the writing down that they were heard, then every new discovery is preceded by the *caveat* of some previous listener.

But if the discovery of a sound consists in separating it from other sounds, and in assigning to it its true place in the history of disease; in ascertaining its causes and true value; and in enabling succeeding observers to draw from it indications for diagnosis, prognosis, and treatment, then is the author of the Prize Essay entitled to the little that he claims, viz., not to have been the first to notice, but the first to explain these sounds—not the first to hear a foreign language, but the first to understand and interpret it.

We turn now to Walshe, and shall take occasion to examine *all* that he has said relative to the pitch of thoracic sounds.

The first mention he makes of it, is on pp. 57 and 58 of his work on the Heart and Lungs. We quote, and the reader will notice his opinion as to the identity of intensity and pitch, assumed by the Dublin Press.

"This is sometimes, but not always, correct; for there is, in point of fact, as intense noise in many so called dull, as clear sounds. It is not in *intensity* that the difference which impresses the ear consists, but in duration and in pitch: so long as they both last, one is as intense as the other. Hence it would appear that what is practically called clearness signifies continuousness of sound; and dullness, non-continuousness. * * * * * * Under certain circumstances it becomes possible, even, to assign rudely the pitch of the percussion-sounds of the chest,—they become notes."

Nothing is here said concerning the practical application of these sounds. We are left in ignorance as to anything farther than the mere fact, that "under certain circumstances" which we are left to divine for ourselves, "it becomes possible, even, to assign *rudely* the pitch of the percussion-sounds."

After considerable search in the volume of Dr. Walshe, we are unable to find any distinct reference to pitch in percussion-sounds, other than that quoted, except the following:

"So, again, equal portions of heart-substance and of liver-substance, when similarly percussed, will give out sounds, short, abrupt, and toneless, in no wise distinguishable from each other; yet the heart and the liver, *in situ*, sound differently: the pitch of the heart sound is perceptibly higher than that of the liver, and the difference depends on the hollow form of the former, plus the different properties of the cavities containing the two organs."

This, while correct in point of fact, would never have been explained by the "hollow form of the organ," had Dr. Walshe been familiar with the principle laid down in the prize essay of Dr. Flint:

"*An elevation of pitch always accompanies diminution of resonance in consequence of pulmonary consolidation.*"

Had the hollow form of the heart any influence upon its pitch, the pitch would be lowered instead of elevated. But the facts are, evidently, that the greater density of the walls of the heart, and the presence in its cavity of an unelastic, incompressible fluid, give to it its elevated pitch as compared to the liver, and that the hollow form of the organ has no influence whatever.

This explanation of the pitch of the heart-sound is, to us, sufficient evidence that Dr. Walshe, who is so confidently quoted by the "Press," knew little of pitch save its existence; while its application to diagnosis was unseen, and its true philosophy evidently confused in his mind. To this conclusion we are forced by the fact, that in the whole of the remainder of the article on "percussion," Dr. Walshe makes no mention whatever of pitch.

We find, however, under the head of auscultation, the following distinct mention of pitch of respiratory sounds:

"The *pitch* of sounds rises as the frequency of the vibrations in a given time of the sonorous body; the evident variations in pitch of the respiratory murmurs under different circumstances immediately depend on variations in that frequency—but why, or through what mechanism, the frequency is affected by different anatomical conditions is unknown."

In speaking of the blowing sound, Dr. Walshe states that both inspiratory and expiratory sounds are of a higher pitch than natural.

The reader of the Prize Essay will recollect, that it is distinctly and prominently stated that in the sibilant and sonorous râles, the distinction as to pitch had been previously made, while Barth, and Roger, mention elevated pitch as a quality of bronchial sounds.

But the question is concerning percussion-sounds especially, though it might be extended to auscultatory sounds without impeding to any extent the tenor of our argument. For, in all works on auscultation-sounds, pitch is mentioned in so trivial and unsatisfactory a manner, that they afford no light upon the value of the sign.

We continue our quotations from the "Press":

"Besides we had, years back, the elaborate monograph by Fournet, which was the first to point out the existence of an expiratory murmur in all forms of respiration, and which puts in the clearest light the great importance of attending to this particular murmur both in health and in disease, and more particularly as a diagnostic of much value in the very earliest stage of phthisis, the point to which nearly the whole of this essay before us is devoted. We must take it for granted that our author has never seen this work; and hence as has occurred in so many other instances, his observations are, to a certain extent, original. We might allude likewise to the very able work by Skoda, which, however, it is possible, may not yet have reached America. It contains much bearing on the subjects touched upon in the essay."

We are ignorant of the "monograph by Fournet." He could scarcely, however, have been the first to point out the existence of an expiratory murmur, since that is a self-evident fact—it requires no *savant* to prove that *every* inspiratory must, of necessity, be followed by an expiratory murmur. If, however, Fournet has advanced anything relative to the *pitch* of the expiratory murmur, his monograph has some bearing on the subject. But the Press does not assert any such knowledge on the part of Fournet.

It is not, in any sense, claimed that Dr. Flint was the first to notice the expiratory murmur, or its importance in incipient phthisis. It does not occupy the "large part of the essay" assigned to it by the "Press," and it only mentions it, as it does œgophony, or any other well known sound, except to give it its due importance as an early symptom of pulmonary obstruction. We can, of our own knowledge, assure the "Press" that Dr. Flint has not only seen Skoda's work, but has actually perused it. That "it contains much bearing on the subject touched upon in this essay" is indisputable, and is equally true of all other works on physical diagnosis.

We finish with the following extract from the article of the "Press":

"At page 12 we have a point started by the writer, and one of much consequence to determine, we mean whether there be any natural difference in the strength of the bronchial murmur on the right and left sides. The writer says that on the right side the pitch was found to be distinctly higher than upon the left; in the proportion of three to one. He goes on then to state that Dr. Stokes, of Dublin, has arrived at the very opposite result, for he has made the pitch highest on the left side. Our author has here fallen into a mistake, for Dr. Stokes was not speaking of the bronchial respiration at all but of the vesicular, which, he says, is, in the majority of instances, distinctly louder at the apex of the left than the right lung. This point, if true, is one of much consequence. We believe, however, it requires further investigation."

The secret of this apparent difference of opinion, lies in the fact that the Dublin editor repeats here the error with which he started, and which he maintains with unwavering consistency. He does not recognize any difference between intensity and pitch, considering two very different qualities of sound as identical.

Again, Dr. Flint does not contradict Dr. Stokes. He reconciles a discrepancy between Stokes and Gerhard, by proving that both were right; in a word, that Stokes spoke of the murmur as *louder* on the left side, while Gerhard and Flint found it *higher as to pitch* on the right side. Both conditions might exist in the same patient.

We should not have given this notice so formal a consideration, were it not that our Americanism is somewhat interested in it. The cool assumption of higher attainment, the nonchalance with which European writers appropriate to themselves American discoveries, and the unwillingness with which they concede to transatlantic students the credit of their investigations, contrast so strongly with the liberality, and almost sycophantic eagerness with which European authorities are received by our medical public, that we confess to a feeling somewhat akin to indignation.

It happens, too, that the universal voice of medical opinion upon this side, had conceded some degree of originality to the prize essay in question.

In conclusion, we need only say that this reply to the criticism of the "Dublin Medical Press" is written without any knowledge of it on the part of the senior editor, and that the junior is alone responsible for its sentiments.

